# Mitochondrial dysfunction and DNA damage accompany enhanced levels of formaldehyde in cultured primary human fibroblasts

**DOI:** 10.1038/s41598-020-61477-2

**Published:** 2020-03-27

**Authors:** Cristina A. Nadalutti, Donna F. Stefanick, Ming-Lang Zhao, Julie K. Horton, Rajendra Prasad, Ashley M. Brooks, Jack D. Griffith, Samuel H. Wilson

**Affiliations:** 1 Genome Integrity and Structural Biology Laboratory, National Institute of Environmental Health Sciences, NIH, Research Triangle Park, NC 27709 USA; 2 Center for Integrative Bioinformatics, National Institute of Environmental Health Sciences, NIH, Research Triangle Park, NC 27709 USA; 3 Lineberger Comprehensive Cancer Center, University of North Carolina at Chapel Hill, Chapel Hill, NC USA

**Keywords:** Mitophagy, Double-strand DNA breaks

## Abstract

Formaldehyde (FA) is a simple biological aldehyde that is produced inside cells by several processes such as demethylation of DNA and proteins, amino acid metabolism, lipid peroxidation and one carbon metabolism (1-C). Although accumulation of excess FA in cells is known to be cytotoxic, it is unknown if an increase in FA level might be associated with mitochondrial dysfunction. We choose to use primary human fibroblasts cells in culture (foreskin, FSK) as a physiological model to gain insight into whether an increase in the level of FA might affect cellular physiology, especially with regard to the mitochondrial compartment. FSK cells were exposed to increasing concentrations of FA, and different cellular parameters were studied. Elevation in intracellular FA level was achieved and was found to be cytotoxic by virtue of both apoptosis and necrosis and was accompanied by both G2/M arrest and reduction in the time spent in S phase. A gene expression assessment by microarray analysis revealed FA affected FSK cells by altering expression of many genes including genes involved in mitochondrial function and electron transport. We were surprised to observe increased DNA double-strand breaks (DSBs) in mitochondria after exposure to FA, as revealed by accumulation of γH2A.X and 53BP1 at mitochondrial DNA foci. This was associated with mitochondrial structural rearrangements, loss of mitochondrial membrane potential and activation of mitophagy. Collectively, these results indicate that an increase in the cellular level of FA can trigger mitochondrial DNA double-strand breaks and dysfunction.

## Introduction

Genome stability is important for maintenance of normal cellular metabolism and is essential for cell survival. During evolution, cells have developed highly conserved mechanisms of DNA repair to prevent genetic alterations due to DNA damage caused by endogenous as well as exogenous sources^1,2^. Eukaryotic cells contain two genomes, nuclear and mitochondrial (mtDNA), and it is suggested that oxidative stress in mitochondria will also impose oxidative stress on the nucleus^3–5^. Therefore, oxidative stress may impact both genomes in a highly interconnected fashion.

Mitochondria are double membrane organelles and generate most of the cell’s supply of adenosine triphosphate (ATP), used as a source of chemical energy for cellular functions by establishing intricate genetic interactions with the nuclear compartment. However, among the approximately 1,200 proteins needed for mitochondrial metabolism, only a few are encoded by the mitochondrial genome^6^. The mammalian mitochondrial DNA is a double-stranded, closed-circular molecule assembled into compact structures called nucleoids. The mitochondrial genome encodes 13 proteins that are key components of the oxidative phosphorylation system (OXPHOS), in addition to 2 rRNAs and 22 tRNAs, necessary for the mitochondrial translational machinery^7^. OXPHOS is characterized by multimeric complexes, that are responsible for the transfer of electrons to molecular oxygen, leading to protons being pumped into the inter-membrane space, and ultimately to complex V (also known as ATP synthase), for the generation of ATP. Many reactive molecules are produced during OXPHOS in the mitochondrial matrix and the majority are formed when electrons leak from the electron transport chain and interact with molecular oxygen creating reactive oxygen species (ROS). ROS oxidize membrane lipids to generate aldehydes, which in turn chemically modify DNA bases that become abundant in aging and diseased human tissues. Thus, compared to the nuclear DNA, mtDNA is up to 100 times more vulnerable to damage^8,9^. This vulnerability is exacerbated by the absence of an efficient nucleotide excision repair system (NER), such that mitochondria appear to lack the ability to repair certain helix-distorting lesions induced in mtDNA by environmental genotoxins and endogenous metabolites^10–13^. Therefore, the mtDNA lesions produced, if not repaired and/or eliminated by mitophagy, can affect mtDNA replication and transcription, leading to mitochondrial dysfunction^14–17^.

During the last decades, increasing research has focused on formaldehyde (FA), a naturally occurring organic compound, and on its effects on human health. The National Toxicology Program listed FA as a potential human carcinogen in 1981, based on evidence from studies in experimental animal models. Since then, several additional cancer studies have been published, and FA was classified as a human carcinogen in 2006^18,19^. FA covalently binds to DNA and forms DNA monoadducts, DNA-DNA interactions, DNA-protein and DNA-glutathione crosslinks^20^ that can lead to genotoxic stress if not removed^2,21,22^. In humans, the enzymes alcohol dehydrogenases (ADH2/ADH5), the glutathione system, and the Fanconi anemia group D2 protein (FANCD2), a DNA crosslink repair protein, provide defense mechanisms against potential genotoxic effects caused by FA^21,23,24^.

Until recently, it was believed that small amounts of FA in bodily fluids of healthy people were derived mainly from consumption of alcoholic beverages. However, recent data have shown that FA naturally occurs as a normal byproduct of metabolism^25,26^, and altered levels of FA may correlate with adverse conditions, such as cancer and neurodegenerative disorders^27–30^. The full range of physiological roles of FA is still unclear, however, as are the consequences of FA accumulation within biological systems.

There have been somewhat limited investigations connecting intracellular FA metabolism with physiology^24,27,29,31–35^. In the present study, we aimed to investigate how primary foreskin (FSK) fibroblast cells responded after exposure to increasing concentrations of FA, especially with regard to the mitochondrial compartment. The results provide insight into the biological processes that modulate FA effects, *in vivo*. We utilized human FSK cells in order to provide a physiological model to study the endogenous deregulation of FA and to investigate the attendant metabolic adaptations.

## Material and Methods

### Cell culture

Primary cultures of human fibroblasts (FSK) were cultured in Dulbecco’s modified Eagle’s medium (DMEM) (Gibco, Carlsbad, California), supplemented with 10% FBS (Hyclone, Fisher Scientific, Canada, USA), 100 nM non-essential amino acids (Gibco) and 50 μg/ml of penicillin/streptomycin in a humidified incubator at 37 °C in an atmosphere of 5% CO_2._ Mycoplasma testing was performed using a MycoAlert® Mycoplasma detection kit (Lonza, Rockland, ME). The fibroblast cells were placed in culture at the Lineberger Comprehensive Cancer Center of the University of North Carolina at Chapel Hill (UNC) by members of the laboratory of Dr. J. Griffith. All of the experimental protocols used in the production of the FSK cells were approved by the UNC Institutional Review Board (01-0705). The FSK cells used in the current study are leftover samples from the previously IRB-approved work, and the FSK cells were stored frozen. Since the cells were primary cultures, they were used only until passage 6. The methods for the use of FSK cells were under guidelines and regulations of the National Institute of Environmental Health Sciences, National Institutes of Health. Human participants were not involved in the present study, and human tissue was not used.

### Determination of cell viability

Cell viability of FSK cells was determined by a crystal violet (CV) assay, as previously described^32^, with minor modifications. Briefly, cells were plated on 6-well plates at the density of 1 × 10^5^ cells per well. At 80% confluence, the cells were exposed to 250 μM or 500 μM FA, for 24 h, 48 h and 72 h and then stained with a solution of CV in 50% methanol (Met-OH) for 1 h at 25 ^o^C. Cells were washed three times with water and CV dye retained by viable cells solubilized in 100% Met-OH. Subsequently, 100 μl of CV solubilized cells were transferred into a 96-well plate and the absorbance was read with a microplate reader at 570 nm (Biotek Synergy Multi-mode microplate reader, Winooski, VT, USA). The fluorescence intensity was taken as proportional to the number of viable cells. The results were expressed as percentage of control cells without any treatment. Average values were calculated from n = 3 wells in each study group. All experiments were performed with different cell batches and repeated at least three times.

### Evaluation of endogenous levels of FA

The endogenous levels of FA in FSK cells under different experimental conditions were determined with a fluorometric FA assay (Sigma) according to the manufacturer’s instructions. Briefly, 25 × 10^6^ cells for each study group were washed three times with phosphate-buffered saline (PBS), collected by trypsinization and lysed as described previously^36^. Proteins in the lysate samples were precipitated by trichloroacetic acid (TCA) solution using 100 μl of cell lysate, and combined with 25 μl of neutralizer (provided by the manufacturer). A sample from the resulting preparation (50 μl) was mixed with the detection solution, and FA was measured after incubation in the dark for 30 min at 25 ^o^C. Absorbance was measured at λ_emiss_ = 370 nm and λ_excit_ = 470 nm (Biotek Synergy Multi-mode microplate reader). The concentration of FA in the samples was calculated using a standard curve. Average values were calculated from n = 3 wells in each study group. All experiments were repeated three times using different cell batches.

### Quantification of apoptotic cells

The number of FSK cells undergoing apoptosis and/or necrosis after chronic exposure to 250 μM and 500 μM FA was quantified using an Annexin V-FITC kit (Trevigen, Gaithersburg, MD, USA), according to the manufacturer’s instructions. Briefly, 1 × 10^4^ cells under different experimental conditions were collected by trypsinization, washed in PBS, resuspended in binding buffer, and mixed with Annexin V-FITC and propidium iodide (PI). After a 15 min 25 ^o^C incubation in the dark, the cells were analyzed using a flow cytometer LSR II flow cytometer (BD Biosciences LSRFortessa, San Jose, CA, USA) and analyzed using Facsdiva software (BD Biosciences). Each experiment was performed in duplicate, repeated at least three times using different cell batches.

### Flow cytometry analysis of cell cycle

For cell cycle analysis, FSK cells, under different experimental conditions, were collected by trypsinization, washed with PBS and stained with bromodeoxyuridine (BrdU) for 2 h and PI for 30 min. An LSR II flow cytometer was used to read samples, which were analyzed using BD Facsdiva software. Each experiment was performed in duplicate, repeated at least three times using different cell batches.

### RNA isolation

FSK cells were seeded in 145-mm dishes at 1 × 10^6^ cells/dish and cultured to 80% confluence. Cells were then treated with 250 μM FA for 24 h and washed twice in PBS. Total cellular RNA was isolated using an RNeasy Midi Kit (Qiagen, TX, USA) according to the manufacturer’s instructions. Genomic DNA was removed by on-column digestion with RNase-free DNase I (Qiagen) and denaturing formaldehyde/agarose gel electrophoresis was performed to validate the quality and integrity of the RNA samples. The samples were quantified using a Nanodrop ND-1000 spectrophotometer (Thermo Scientific, DE, USA), and the purity was assessed by 260/280 absorbance. Three biological replicates were collected and isolated from control and 250 μM FA-treated cells.

### Microarray

Gene expression analysis was performed using Agilent Whole Human Genome 4 × 44 multiplex format oligo arrays (Agilent Technologies, CA, USA) following the Agilent one-color microarray-based gene expression analysis protocol. Starting with 500 ng of total RNA, Cy3-labeled cRNA was produced according to the manufacturer’s protocol. For each sample, 1.65 μg of Cy3-labeled cRNA was fragmented and hybridized for 17 h in a rotating hybridization oven. Slides were washed and then scanned with an Agilent Scanner. Data were obtained with Agilent Feature Extraction software (v.9.5), using the one-color defaults for all parameters, performed error modeling, adjusting for additive and multiplicative noise. The resulting data were processed using OmicSoft Array Studio software (v.7.0). Significant probes were determined by filtering data to include only probes with fold changes >1.5 or <1.5 compared with the control and adjusted p-values < 0.01, which was determined by an error-weighted one-way analysis of variance (ANOVA) and Bonferroni multiple test correction using the OmicSoft software. Further, Gene set enrichment analysis (GSEA)^37^ was performed to identify potential functional enrichment. The curated canonical pathway gene sets (C2:CP, version 7) in Broad Molecular Signature Database (MSigDB) were used to determine enriched pathways. Gene sets with FDR < 0.25 were considered as significantly enriched. The microarray expression data has been submitted to GEO.

### Sample preparation for electron microscopy

FSK cells, untreated and treated with 250 μM FA for 24 h, were collected, fixed for 1 h at 25 ^o^C in freshly prepared 2.5% glutaraldehyde (Electron Microscopy Sciences, Hatfield, PA, USA) and post-fixed with osmium tetroxide 1% (Electron Microscopy Sciences). Cells were then dehydrated in a graded series of ethanol solutions before the embedding in EPON-812 Resin (Electron Microscopy Sciences) and allowed to polymerize for 48 h at 60 ^o^C. Using a Leica EM UC7 Ultramicrotome, ultra-thin sections of 50–60 μm were obtained with a diamond knife and collected either on copper or Nickel (Ni) grids. For cell ultrastructural analysis some specimens were counterstained with 2% uranyl acetate and lead citrate. At least 100 images for each study group were captured using a Gatan Orius real time CCD camera (Pleasanton, CA, USA) attached to an FEI Tecnai T12 TEM/STEM instrument (Hillsboro, OR, USA) operated at 80 kV. Electron microscopy (EM) subcellular analysis was performed using Gatan Digital Micrograph software.

### Mitochondrial membrane potential

FSK cells were seeded on 145-mm dishes at 1 × 10^6^ cells/dish and exposed to 250 µM FA for 24 h. The mitochondrial membrane potential (ΔΨm) in control and treated cells was assessed with the Mitochondrial Membrane Potential Assay Kit (II) (Cell Signaling Technology, Danvers, MA, USA) by flow cytometry. Carbonyl-cyanide-3-chlorophenylhydrazone (CCCP) at 25 µM final concentration was used as positive control to induce ΔΨm loss. All samples were subsequently labeled with 2 µM tetramethylrhodamine ethyl ester (TMRE) for 30 min and Sytox Blue was added just prior to examination to exclude dead cells. Cells were examined using an LSR II flow cytometer and analyzed using Facsdiva software. Each experiment was performed in duplicate, repeated at least three times using different cell batches.

### Flow cytometry analysis of double-strand DNA breaks

In order to characterize the extent of DNA damage induced by chronic exposure to FA, FSK cells were seeded 1 × 10^6^ on 145-mm dishes and treated for 24 h with 250 μM FA. Thereafter, cells were collected, washed in PBS and prepared for flow cytometry using the H2A.X Phosphorylation Assay Kit for flow cytometry (EMD Millipore) according the manufacturer’s instructions. Briefly, cells were collected, stained and resuspended in 500 µl of PI in the dark for 30 min. Samples were read on an LSR II flow cytometer and analyzed using Facsdiva software. Each experiment was performed in duplicate, repeated at least three times using different cell batches.

### Immunofluorescence (IF) stainings

FSK cells, 5 × 10^5^, were cultured on 35 mm glass bottom dishes (MatTek Corporation, MA, USA) for 24 h in the presence and in the absence of 250 μM FA. The cells were then washed, fixed with 4% paraformaldehyde (PFA), blocked with 3% BSA (Sigma-Aldrich) and permeabilized with 0.25% Triton X-100 (Sigma-Aldrich). Immunolabeling was carried out using anti-TOMM20 Alexa Fluor® 647-conjugated (1:500), anti-53BP1 Alexa Fluor® 488-conjugated, anti-PINK1 FITC-conjugated (Abcam). CCCP at 1 µM final concentration was used as positive control for the recruitment of PINK1 to the mitochondria. DNA was visualized using an anti-DNA primary antibody (Abcam) (1:200) with IgM-PE as secondary antibody (1:1000). DNA DBS breaks were also monitored using a phospho-H2AX (S139) FITC-conjugated antibody and etoposide (Sigma) at 50 nM final concentration was used as positive control for inducing DNA DBSs in the mitochondrial DNA. Nuclei were counterstained with NucBlue® Fixed Cell Stain Ready Probes™ (Life Technologies) and all IF images were acquired with 60× oil immersion objective on the Zeiss LSM780 controlled by Zen 2012 SP2 software (Carl Zeiss MicroImaging). Each staining was performed in duplicate and repeated two times using different cell batches.

### Statistical analysis

All the data were statistically analysed with the non-parametric two-tailed Mann-Whitney U test and presented as means ± S.E.M. Results were considered significant at p < 0.05.

## Results

### Human primary fibroblasts are sensitive to FA exposure

**Figure 1:**
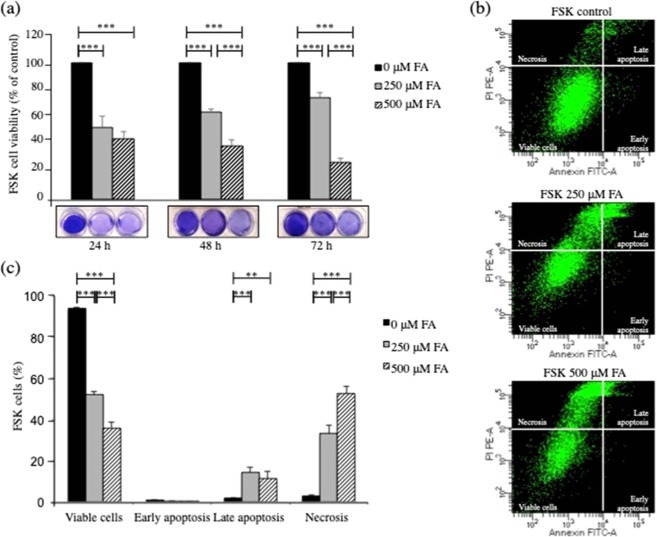
FA exerts cytotoxic effects in human primary fibroblasts. (**a**) Crystal violet cell viability assay of FSK cells without and in the presence of 250 μM and 500 μM FA for 24 h, 48 h and 72 h. The cells showed different sensitivity to 250 μM FA and 500 μM FA treatments. Treatment of the cells with 250 μM FA was less harmful than exposure to 500 μM FA, that was highly cytotoxic at all the time points considered. The results are expressed as percentage of control cells without any treatment. Average values were calculated from n = 3 wells in each study group. All experiments were performed with different cell batches and repeated at least three times. The fluorescence intensity was taken as proportional to the number of viable cells. Statistical analysis was performed using the non-parametric two-tailed Mann-Whitney U test. Data are presented as means ± S.E.M. A p value < 0.05 was considered significant. (**b**) Flow cytometry representative images of Annexin V and PI staining and (**c**) relative quantification. FA induced apoptosis and necrosis in a dose dependent manner. After treatment with 250 μM and 500 μM FA for 24 h, the cells showed increased apoptosis and necrosis at rates of 14.5%, 33.5% and 11%, 53% respectively. Data were expressed as percentage of control cells and the bars represent the mean and error bars S.E.M. A p value ≤ 0.05 was considered significant (*p ≤ 0.05, **p ≤ 0.01, ***p ≤ 0.001).

To gain insight into the *in vivo* effects of FA, we studied the cytotoxic effects of exposure to FA in human primary foreskin (FSK) cells. Based on results from range-finding assays, we focused initially on FA concentrations of 250 μM and 500 μM. The effect of exposure to 250 μM FA was a drop in cell viability (to 47%) after 24 h, with a partial recovery after 48 h and 72 h (Fig. 1a). The cells were more sensitive to 500 μM FA at each time point. Since analysis revealed that FA treatment caused cell death, we assessed apoptosis and necrosis by flow cytometry using double labeling with Annexin V and PI (Fig. 1b,c). The number of apoptotic cells increased after treatment with FA for both concentrations employed in the study (Fig. 1b,c). In addition, we observed an increment of necrotic cells after 24 h treatment with 250 μM and 500 μM FA (Fig. 1c).

FA is present in the human bloodstream at a concentration of about 20–100 μM^38–40^. In the current study, to provide a measurement of endogenous FA, we determined the levels of FA before and after exposure to 250 μM FA (Fig. S1a–c). Addition of 250 μM FA in the culture medium of FSK cells for 24 h increased the intracellular level of FA about 2-fold compared to untreated (control) cells (Fig. S1c).

### Increased intracellular FA induces cell cycle changes in human primary fibroblasts

**Figure 2:**
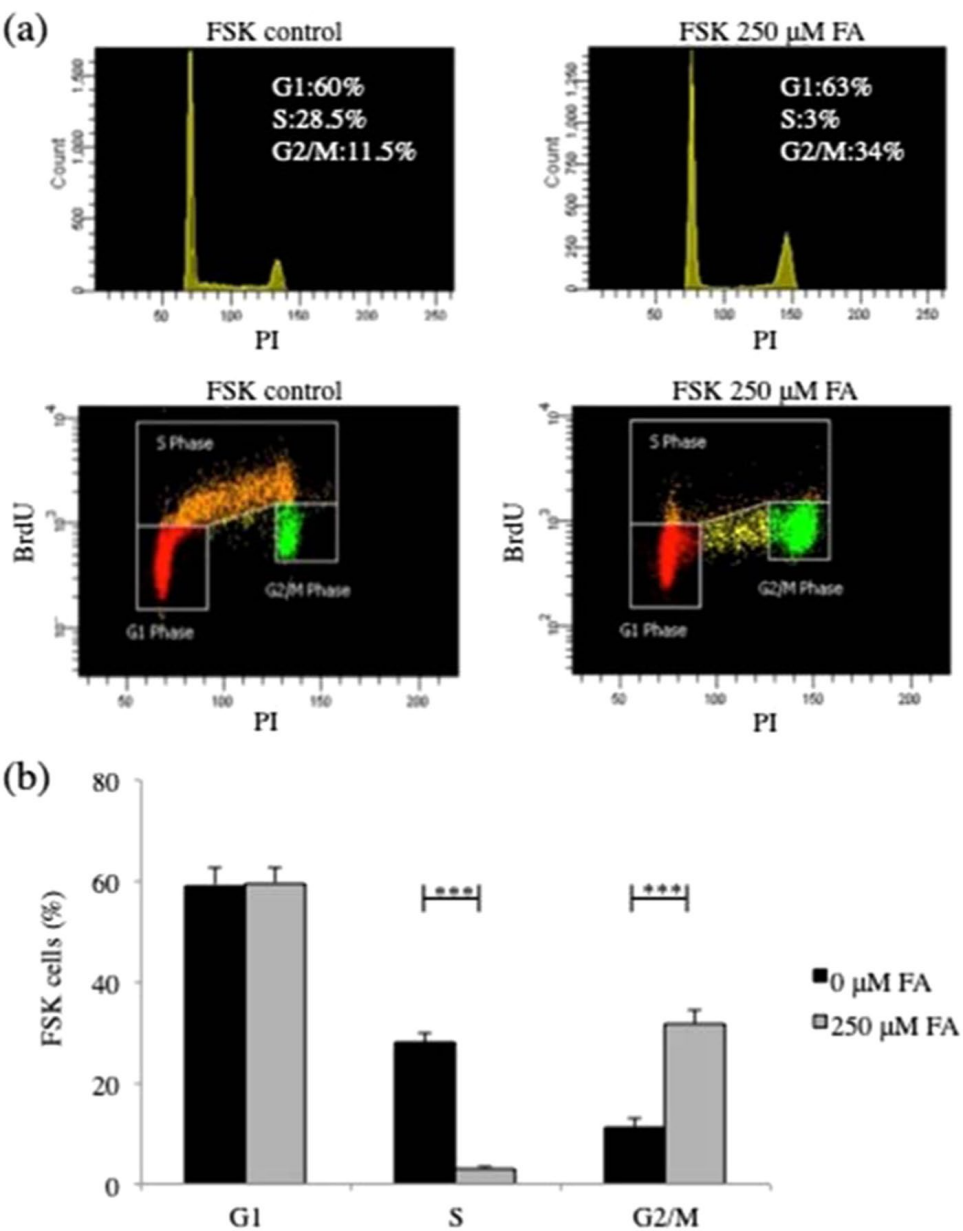
Increased intracellular FA induced G2/M DNA-damage cell cycle arrest in human primary fibroblasts. (**a**) Representative flow cytometry images of cell cycle analysis in FSK cells in the presence and in the absence of 250 μM FA for 24 h and (**b**) relative quantification. The cells were collected, stained with BrdU and PI, and analysed for cell cycle distribution by flow cytometry. All experiments were performed with different cell batches in duplicate and repeated at least three times. (**b**) FA increased the proportion of cells in G2/M phase and decreased that in the S phase. Data are expressed as percentage, and in all panels, the bars represent the mean and error bars S.E.M. A p value ≤ 0.05 was considered significant (*p ≤ 0.05, **p ≤ 0.01, ***p ≤ 0.001).

To determine whether changes in the cell cycle were associated with the decrease in cell viability observed after FA treatment of FSK cells, we conducted cell cycle analysis in the presence and in the absence of 250 μM FA for 24 h. Incorporation of 5-bromo-deoxyuridine showed that the proportion of cells in G2/M phase was about 12% in control cells (Fig. 2a,b) and 34% with FA exposure (Fig. 2a,b). Thus, the FA-treated cells accumulated in G2/M phase as compared with the control cells (Fig. 2). In addition, while the percentage of cells in S phase in untreated cells was about 30%, this decreased to 3% after FA treatment (Fig. 2a–c), suggesting the absence of *de novo* DNA synthesis.

### Increased intracellular FA alters mitochondrial gene expression as measured by microarray analysis

**Figure 3:**
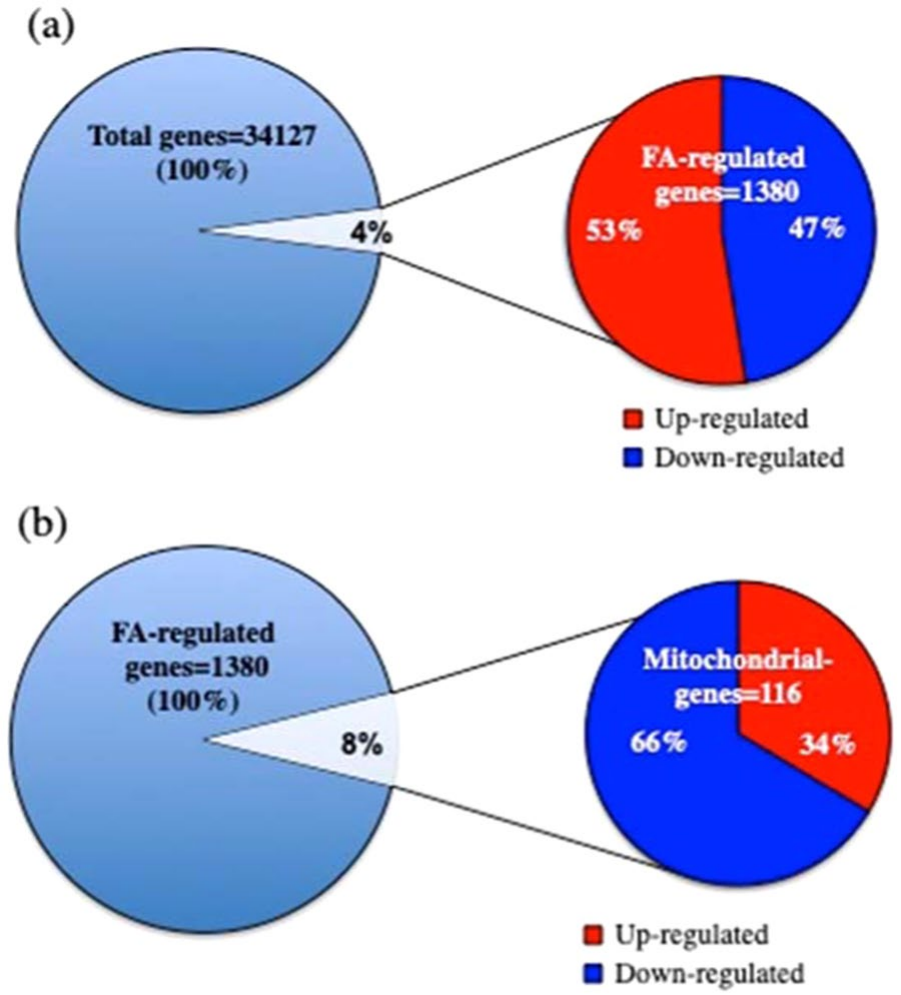
Increased intracellular FA affected gene expression in human primary fibroblasts. (**a**) Total RNA from FSK cells before and after treatment with 250 μM FA for 24 h was used to perform Agilent Whole Human Genome (4x44k) array analysis. (**a**) The down- and up-regulated genes are shown in blue and in red, respectively. All the data were generated from the average of three biological replicates for each study group, and significant probes were identified by selecting those probes showing a *p* value < 0.01, as determined by error-weighted ANOVA with Bonferroni multiple-test correction. (**b**) Increased intracellular FA affected mitochondrial-gene expression in human primary fibroblasts. Among the total genes significantly regulated by FA, 8% were found to be mitochondrial related. The mitochondrial down- and up-regulated genes are shown in blue and in red, respectively.

We examined changes in gene expression in FSK cells after exposure to 250 μM FA for 24 h (Fig. 3 and Table S1). Total RNA from untreated and treated cells was used to conduct Agilent Whole Human Genome (4x44k) array analysis. Genes were considered significant at adjusted *p* value <0.01, as determined by error-weighted ANOVA with Bonferroni multiple-test correction. Across all 34,127 genes studied, 1,380 were FA-regulated with 53% of the genes up-regulated and 47% down-regulated (Fig. 3a). The GSEA software was applied to identify canonical pathways enriched in FA-treated cells. Interestingly, GSEA analysis revealed that FA treatment is associated with impairment of pathways involving immune response (Nuclear factor of activated T cells, NFAT), T cell metabolism (T-cell receptor calcium, TCR-calcium), cellular and developmental response to hypoxia (Hypoxia-inducible factor 1 alpha, HIF-1), cell differentiation, apoptosis and autophagy (p38 mitogen activated protein kinases, p38MAPK), and stress response (activator protein 1, AP-1). The stress-activated p38MAPK pathway is recognized as a checkpoint regulator that is activated downstream of ATM and ATR upon DNA damage. The networks orchestrated by these kinases in turn affect a variety of important cellular processes including cell cycle arrest, DNA repair, chromatin assembly, transcriptional and post-transcriptional regulation of gene expression, and cell death^41–43^.

Because many of the FA-responsive pathways described above are coordinated with mitochondrial metabolism, we further investigated mitochondrial genes, either mitochondrial or nuclear encoded. Remarkably, expression analysis of human mitochondrial genes revealed that 116 genes (8% of total 1380 FA-regulated genes) were altered by FA treatment (Fig. 3b). These mitochondrial genes coordinate pathways involved in the development and regeneration of axons (Axon guidance), immune response (Adaptive Immune System), metabolism of proteins, developmental biology, tricarboxylic acid cycle, respiratory electron transport, ATP synthesis by chemiosmotic coupling and heat production by uncoupling proteins (Figs. 3b, S2, Table S1). In addition, we found that several genes encoding respiratory chain complexes were significantly changed by FA treatment (Fig. 3b, Table S1), and ND5 was the only mitochondrial encoded gene in this group (Fig. S2).

### Increased intracellular FA induces structural rearrangements and mitochondrial membrane potential loss in mitochondria of human primary fibroblasts

**Figure 4:**
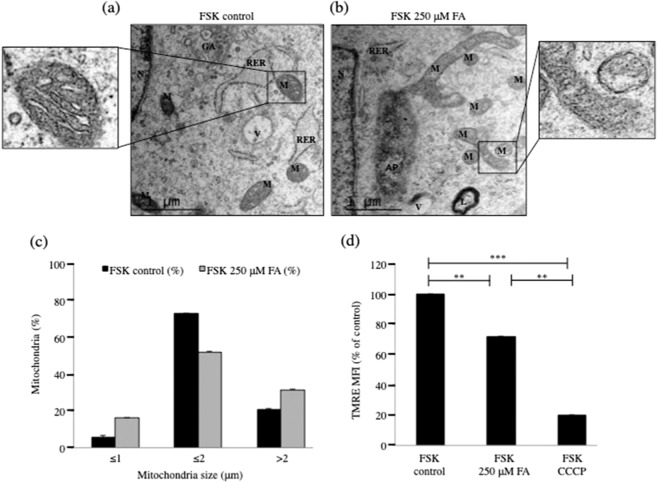
Increased intracellular FA induced structural rearrangements in human primary fibroblasts. Representative EM micrographs of mitochondria from (**a**) untreated and (**b**) FSK treated cells with 250 μM FA for 24 h. Note the narrower, mis-orientated cristae that were reduced in number in FA-treated cells and the smaller mitochondria. At least 200 mitochondria were analyzed for each study group. M = Mitochondrion; RER = Rough Endoplasmic Reticulum; V = Vacuole; L = Lysosome. Scale bar 1 μm. (**c**) Relative size of mitochondria following treatment with 250 μM FA for 24 h. (**d**) Flow cytometry analysis of ΔΨm of FSK cells following the indicated FA treatments. FA induced TMRE loss when compared to parental cells. The mitochondrial uncoupler, carbonyl cyanide m-chlorophenyl hydrazine (CCCP), was employed as a positive control. All experiments were performed with different cell batches in duplicate and repeated at least three times. Data were expressed as mean fluorescence intensity (MFI) percentage of control, and in all panels, the bars represent the mean and error bars S.E.M. A p value ≤ 0.05 was considered significant (*p ≤ 0.05,**p ≤ 0.01, ***p ≤ 0.001).

Because gene expression assessment by microarray showed that FA induced mitochondrial gene alterations, we performed ultrastructure examination of control and FA-treated cells by EM. This was to further investigate the mitochondrial structural rearrangements highlighted by flow cytometry and confocal microscopy. Therefore, using EM and image analysis software, we characterized mitochondrial size with single-organelle resolution (Figs. 4, S7). The circumferences of more than 200 mitochondria were measured in each study group and correlated with the expected mitochondrial size. Mitochondria from control cells contained a dense matrix and well-organized cristae that predominantly oriented transverse to the long axis of the mitochondria (Fig. 4a). About 73% of mitochondria had a perimeter in the range of the expected mitochondrial size of about 2 μm (Fig. 4c), whereas 6% were smaller than 1 μm and 21% larger than 2 μm. In contrast, mitochondria from cells treated with 250 μM FA were morphologically different, with the cristae noticeably thinner and often misoriented and/or decreased in number (Fig. 4b). Only 52% of the mitochondria had a perimeter in the range of controls (2 μm) (Fig. 4c), with an almost 3-fold increase of mitochondria smaller than 1 μm and 1.5-fold increase in mitochondria larger than 2 μm. These mitochondrial structural rearrangements suggest that the enhancement of intracellular FA concentration promoted mitochondrial fragmentation and mitophagy, offering a defense mechanism for maintenance of bioenergetics in the presence of the stress stimulus. Thus, FA exhibits “mitohormetic” effects^44,45^. Interestingly, the treatment of FSK cells with 250 μM FA for 24 h was not coupled with increased mitochondrial fission (Fig. S3).

Mitochondrial fragmentation is associated with loss of mitochondrial membrane potential (ΔΨm). Since ΔΨm is a driving force for mitochondrial ATP generation and ΔΨm loss implicates impaired mitochondrial function, we further investigated ΔΨm in FSK cells untreated and treated with 250 μM FA for 24 h; membrane potential was assessed using the sensing dye, TMRE, that is readily sequestered by active mitochondria (Fig. 4d). Control cells stained with TMRE showed normal polarized mitochondria while FSK cells exposed to 250 μM FA for 24 h, exhibited a significant TMRE loss in comparison to the control group. These results indicated that FA caused a dissipation of ΔΨm.

### FA-induced mitophagy in human primary fibroblasts

**Figure 5:**
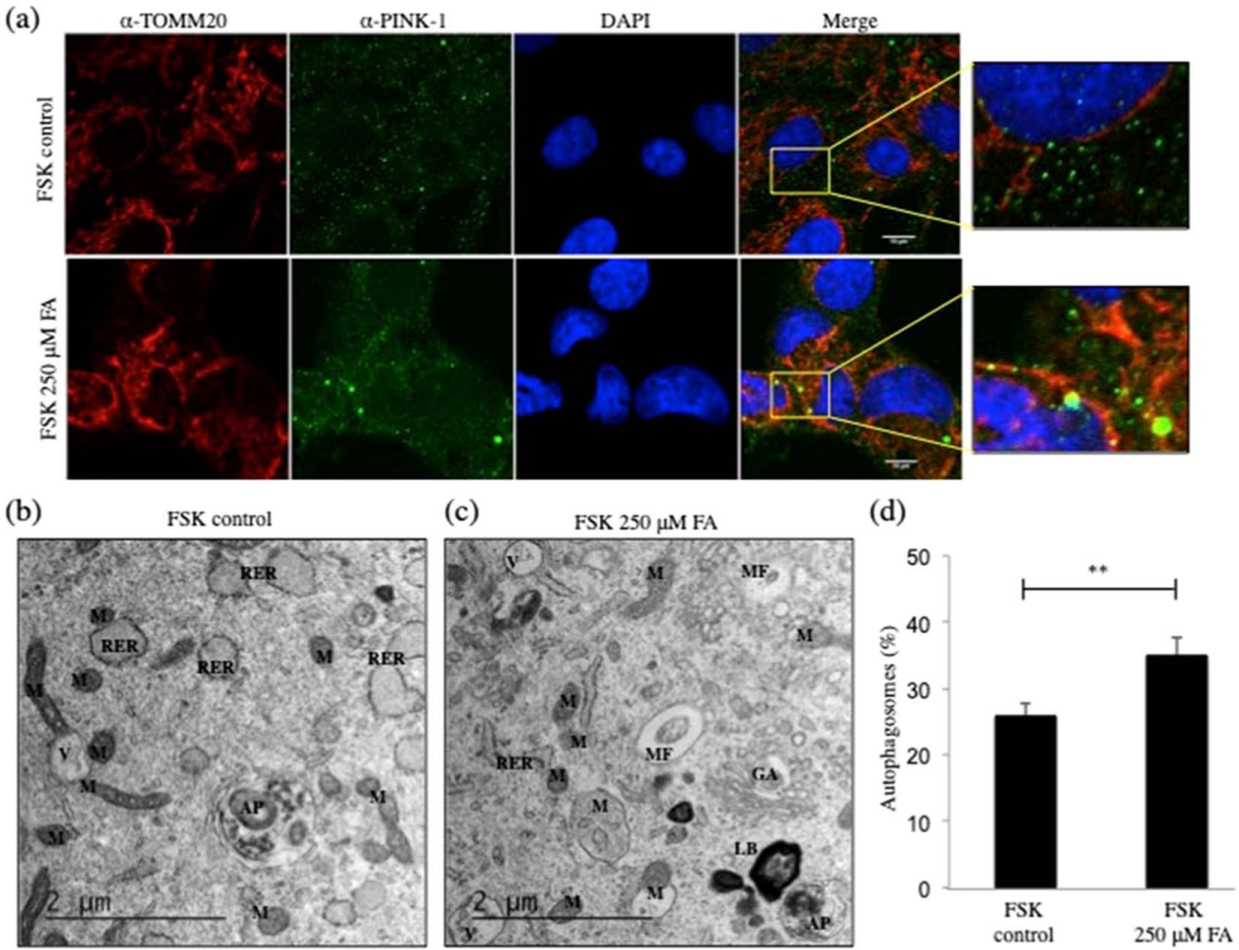
Increased intracellular FA induced selective accumulation of PINK1 in mitochondria of human primary fibroblasts. (**a**) Representative confocal microscopy images of FSK cells following treatment with 250 μM FA for 24 h showing merged channels of mitochondria visualized with TOMM20 (in red) and mitophagy (in green) with PINK-1. The nuclei were counterstained with DAPI (blue). FA-induced accumulation of PINK-1 in mitochondria and the colocalization of TOMM20 and PINK-1 (in yellow) is shown in the merged panel as well as in the magnification yellow boxed aereas on the right. Scale bar, 10 μm. (**b**) Representative EM micrographs of autophagosomes from FSK cells (**b**) untreated and (**c**) treated with 250 μM FA for 24 h. M = Mitochondrion; RER = Rough Endoplasmic Reticulum; V = Vacuole; LB = Lamellar Body; AP = Autophagosome; MF = Mitophagosome. Scale bar 2 μm. (**d**) Relative EM quantitative analysis of autophagosomes in FSK cells following the indicated FA treatments. Note that the mito-phagosomes are increased in number in FA-treated cells. Data were expressed as percentage of control, and in all panels, the bars represent the mean and error bars S.E.M. A p value ≤ 0.05 was considered significant (*p ≤ 0.05, **p ≤ 0.01, ***p ≤ 0.001).

Loss of ΔΨm indicates a mitochondrial function impairment that is a prerequisite for mitophagy activation^46,47^. Thus, mitochondrial depolarization appears to precede the translocation of target proteins that tag the mitochondria for mitophagy, such as PTEN-induced putative kinase 1 (PINK-1). To investigate the mechanism that mitigates the FA-induced effect, mitophagy-autophagy in FSK cells before and after exposure to 250 μM FA for 24 h was addressed (Figs. 5, S8–9). We found that PINK-1 localizes in the cytoplasm (Fig. 5a, right upper panel) and undergoes voltage-dependent proteolysis in polarized mitochondria, but accumulates in the depolarized mitochondria of FA treated cells (Fig. 5a, right lower panel and Fig. S4, using CCCP as a positive control). In addition, EM analysis of autophagosomes showed that there was an increase in the number of autophagosomes in FSK cells, where intracellular FA was augmented by cell growth in 250 μM FA for 24 h (Figs. 5b–d, S9b,c). Autophagosomes are formed and sequester dysfunctional intracellular components, directing them to lysosomes for degradation. This autophagic process is the sole known mechanism for mitochondrial turnover, and it appeared to be enhanced after FA treatment (Fig. 5d).

### Increased intracellular FA induces DNA DSBs in mitochondria of human primary fibroblasts

**Figure 6:**
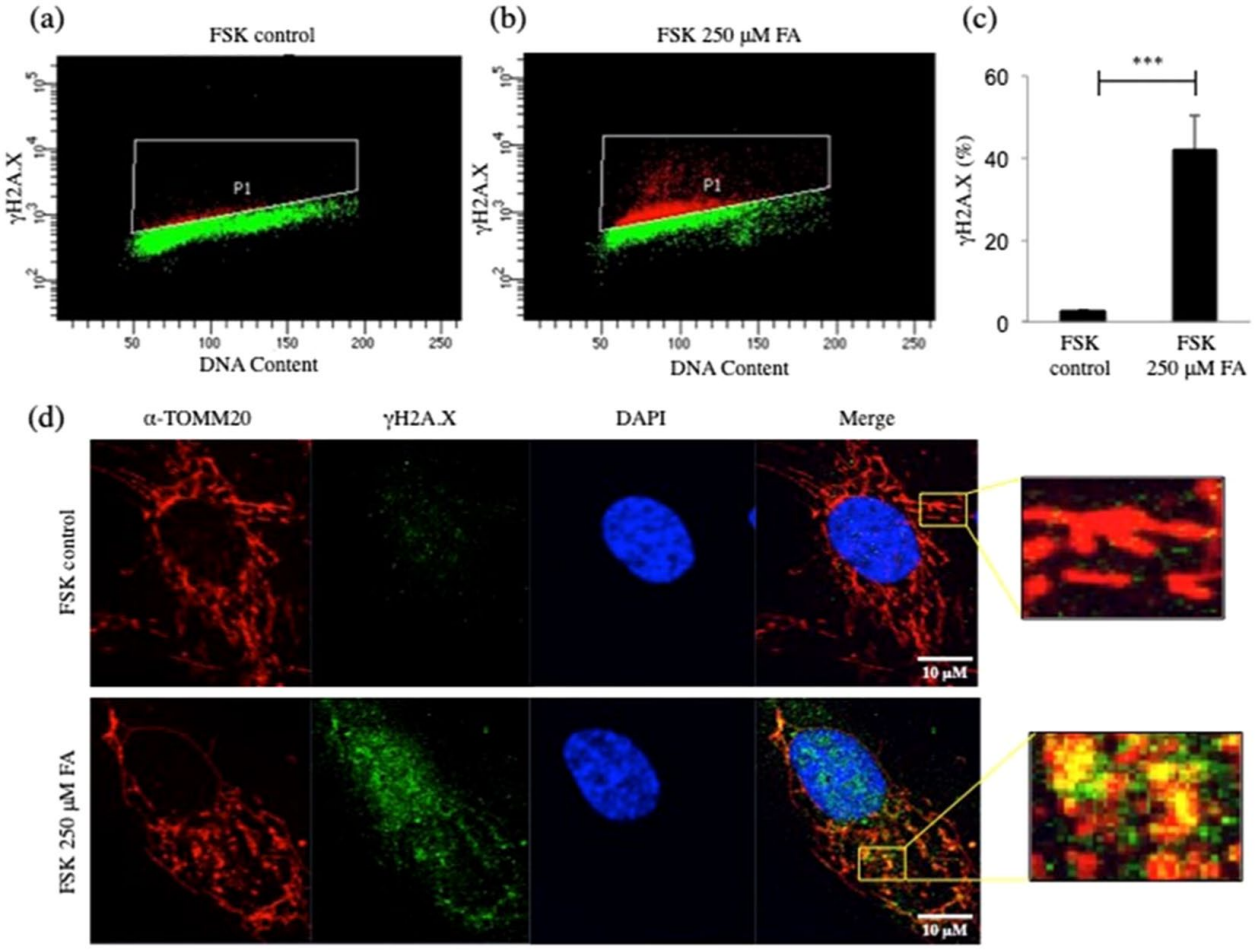
FA-induced-DNA-damage in human primary fibroblasts. (**a**,**b**) Flow cytometry representative images of DNA DSBs in FSK cells with and without 250 μM FA for 24 h and (**c**) relative quantification. The cells were collected and analysed for γH2A.X by flow cytometry using an antibody conjugated with FITC, and DNA content was detected with PI. Treatment with FA increased the proportion of γH2A.X (**b**), when compared to the control group (**a**). (**c**) Quantification of the distribution of γH2A.X in FSK cells in the presence and absence of FA. Experiments were performed in duplicate and repeated at least three times with different cell batches. Data are expressed as percentage, and in all panels, the bars represent the mean and error bars standard error of mean. A p value ≤ 0.05 was considered significant (*p ≤ 0.05, **p ≤ 0.01, ***p ≤ 0.001). (**d**) Representative confocal microscopy images of FSK cells following the indicated FA treatments, showing merged channels of TOMM20 (red) and γH2A.X (green). The nuclei were counterstained with DAPI (blue). Scale bar, 10 μm.

Mitochondrial impairment has been linked to DNA damage and this can induce the phosphorylation of histone H2AX (γH2A.X) on Ser-139 at sites flanking the damage^48^. Formation of γH2A.X at the DSBs site is responsible for recruiting DNA repair proteins to promote repair, and γH2A.X represents a sensitive reporter of DNA damage, in particular of DSBs. The phosphorylation of γH2A.X at DSBs extends up to 1–2 megabases flanking DSBs and allows the immunofluorescence (IF) detection of γH2A.X foci formed at the DSBs. Flow cytometry analysis of γH2A.X allows measurement of the extent of DNA damage in single cells and correlation of damage with the DNA content^49^. We observed that the increased level of FA in FSK cells caused an increase in DSBs when compared to the control group (Figs. 6a–c, S10). This result was confirmed using additional confocal microscopy. Human primary fibroblasts, grown in the presence or absence of 250 μM FA, were stained with antibodies against DNA and 53BP1 (p53 binding protein 1). 53BP1 is a DSBs-responsive protein that promotes repair of DSBs by non-homologous end-joining, while suppressing homologous recombination^50–52^. Accumulation of 53BP1 at the level of mitochondrial DNA was observed after exposure of FSK cells to 250 μM FA for 24 h (Fig. S5).

**Figure 7:**
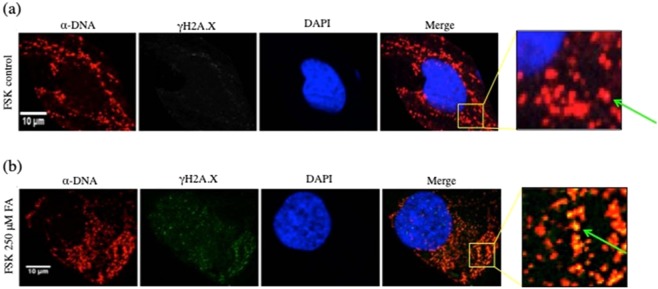
FA-induced-DNA double strand breaks in the mitochondria of human primary fibroblasts. (**a**,**b**) Representative confocal microscopy images of FSK cells following the indicated FA treatments, showing merged channels of DNA (in red) and γH2A.X (in green). The nuclei were counterstained with DAPI (blue). The colocalization of DNA and γ-H2A.X (in yellow) is shown in the merged panel and in the magnification yellow boxed areas with the green arrowheads indicating colocalization in the FA-treated group when compared to control FSK cells. Scale bar, 10 μm.

We further characterized the distribution of DSBs in human primary fibroblasts using confocal microscopy as an alternative method; the localization of DSBs in FA-treated cells was defined at the mitochondrial level (Fig. 6d). Mitochondria, visualized with the import receptor TOMM20, formed a network of long, frequently interconnected, branched tubules in the cytoplasm of control FSK cells (Fig. 6d, upper panel). The network appeared more fragmented and disorganized after treatment with 250 μM FA for 24 h (Fig. 6d, lower panel). When these cells also were probed with γ-H2A.X antibody conjugated with FITC to detect the DSBs (Figs. 7, S6, S11), the protein was observed to be highly localized and clustered at mitochondrial DNA (Figs. 7, S6, S11). Etoposide was used as a positive control (Fig. S6).

## Discussion

FA is a product of natural metabolism that is required in various one-carbon (1-C) addition reactions during biosynthesis of small molecules. Since FA is a reactive chemical capable of forming many adverse side reactions in the cell, it is logical that the FA level must be strictly regulated. Interestingly, an increase of FA in certain types of cancer cells appears to be well tolerated^26,30,33,53,54^. To understand features of FA metabolism in normal cells, we probed the effect of increased levels of FA in primary human fibroblasts (FSK). Conditions for obtaining an increase in FA concentration in FSK fibroblasts in culture were established. The medium was supplemented with FA for 24 h, 48 h and 72 h, and conditions were chosen in this study where the intracellular FA level was increased 2-fold when compared to untreated cells (Fig. S1). This enhanced FA level caused a range of cellular responses, including cytotoxicity (Fig. 1), cell cycle arrest (Fig. 2) and apoptosis (Fig. 1b,c). In addition, gene expression analysis revealed a robust transcriptional response to the increased FA level (Fig. 3, Table S1). The responsive genes were in a number of metabolic pathways (Fig. 3, Table S1), but comprised many genes involved in mitochondrial functions (Fig. 3b), including those involved in the electron transport chain (Fig. S2, Table S1). In light of this, we evaluated mitochondrial properties as a function of intracellular FA. Mitochondrial membrane potential (ΔΨm) was reduced (Fig. 4d) and mitophagy was increased (Figs. 5, S8, S9) with augmented levels of FA, indicating FA-induced mitochondrial dysfunction. Testing of potential DNA damage linked to the FA increased concentration (Fig. 6) revealed surprising results for us: there was a robust induction of DNA DSBs in mitochondrial DNA (Figs. 6d and 7). These results indicate that mitochondria are an important target for FA-induced genotoxicity, and this genotoxicity can be observed with increase intracellular FA level.

### Endogenous FA

FA, a potentially toxic substance in cells, is present in all mammalian cells, yet the adverse effects of a rise in FA intracellular concentration are unknown. There are several processes producing FA in cells that rely on oxidative demethylation of RNA, DNA and proteins, and the oxidative decomposition of certain folate derivates (i.e., dihydrofolate, tetrahydrofolate and 5,10-methylene-tetrahydrofolate^32,55^). The intracellular concentration of FA is maintained homeostatically and is controlled by three major protective systems^29^: glutathione that reacts with FA to form S-hydroxymethylglutathione; the enzyme alcohol dehydrogenase 5 (ADH5) that drives NADP^+^-dependent oxidation of S-hydroxymethylglutathione to S-formylglutathione; and the Fanconi anemia protein complex that performs repair of DNA cross-links mediated by FA. In addition, cellular FA can be reactive, leading to formation of stable methylene bridges with nucleic acids and amines of proteins that ultimately result in crosslinking between nucleic acids and/or proteins. These RNA/DNA-adducts and cross-links are potentially fatal for cells, if not promptly removed.

### Exposure to FA

FA is employed as a preservative, disinfectant, and industrial chemical. Environmental exposures to FA in the workplace and general population are well known. Long-term exposure to FA has been associated with increased risk to several cancer types, cardiovascular disease and neurodegenerative disorders. Although studies have shown that FA exposure can have genotoxicity features, most of these studies considered the effects of FA at a high level, and there is a lack of understanding focusing on FA at lower physiological levels found in cells.

### Elevated intracellular FA

Here, we tested genotoxic properties of elevated intracellular concentration of FA in cultured primary human fibroblasts (FSK). Increased cellular FA was achieved by titrating FA into culture medium of FSK cells (Fig. S1), and higher levels were found to decrease cell viability with an increase in apoptosis and necrosis (Fig. 1). This augmentation of FA concentration also induced G2/M and S phase cell cycle alterations (Fig. 2), and microarray gene expression analyses (Fig. 3) identified many mitochondrial genes regulated by FA (Fig. 3b, Table S1). This is the first whole genome analysis to evaluate the effects of elevated levels of FA in human cells, and this study revealed a strong impact of elevated endogenous FA on mitochondrial metabolism. GSEA showed that FA-regulated genes were involved in immunomodulation, inflammation, stress response as well as energy metabolism. The nuclear factor of activated T cells (NFAT) gene, recently determined to be mitochondrial regulated, was affected by FA^56^. These results suggest that the mitochondrial compartment might be more sensitive to FA-induced toxicity than previously recognized. For this reason, we further investigated the effects of elevated intracellular FA on mitochondria of FSK cells using transmission electron microscopy (EM)^57^ and other methods. The results revealed FA-induced mitochondrial dysfuction by virtue of augmented mitochondrial fragmentation (Fig. 4a–c) and a decline in ΔΨm (Fig. 4d). The partial cristolysis found in FSK cells mitochondria after incubation with FA (Fig. 4b) indicated that the ability of the cells to generate ATP by oxidative phosphorylation had been compromised. It is well established that mitochondrial quality control systems are activated under stress conditions^58,59^. In order to restore the bioenergetics capacity of cells, glycolysis increases and damaged mitochondria are removed through mitophagy/autophagy; this can be detected by mitochondrial fragmentation and lower ATP concentrations^60^. Our results revealed that FA-induced loss of ΔΨm and also triggered cytosolic PINK-1 relocation to the mitochondrial compartment (Fig. 5a). This finding was further corroborated by EM (Fig. 5b–d). Morphometric analysis showed that more autophagosome-like structures were present with increased levels of FA. This was consistent with the induction of mitophagy observed by confocal microscopy (Fig. 5a).

### FA-induced autophagy

DNA repair and autophagy are intimately linked biological processes^61,62^. These processes are essential for maintenance of cellular homeostasis, and when defective, can lead to several adverse conditions. The interchange between the repair and autophagy pathways is complex and highly regulated. Autophagy is activated in response to several types of DNA lesions^63^, and autophagy can regulate different mechanisms and molecules involved in the DNA damage response (DDR), such as induction of cell cycle checkpoints, cell death, and DNA repair. To test the hypothesis that direct activation of mitophagy/autophagy occurs following mtDNA damage induction, we searched for proteins that could be required in this process. We showed that PINK1 removes damaged mitochondria from FSK cells via mitophagy/autophagy. Thus, this represents a mechanism used by these cells to deal with mtDNA DSBs induced by the increase in intracellular levels of FA.

### FA-induced genotoxicity

Our results raised the question of whether DNA damage was linked to the different cellular features characterized. It is known that mitochondrial DNA is far more prone to oxidative injury than nuclear DNA, yet the precise molecular mechanisms that lead to the persistence of lesions in the mitochondrial genome are not well understood^11,64^. However, several explanations have been offered to clarify the vulnerability of mtDNA to damage, including the lack of nucleosome-like structures, as compared to nuclear DNA, limited mitochondrial DNA repair pathways, as well as the proximity of the mtDNA to reactive oxygen species (ROS). To date, the primary well-known sources of mtDNA DSBs are deficient replication, chemotherapeutic drugs, and ionizing radiation, and evidence that an increase of intracellular FA can produce mtDNA DSBs had not been reported.

## Conclusions

We demonstrated a physiologically relevant link between endogenous FA level and mitochondrial DNA damage. Elevation of the cellular level of FA in primary human fibroblasts resulted in accumulation of mtDNA DSBs and perturbation of mitochondrial metabolism. The findings indicate that FA-induced mitochondrial dysfunction stimulates activation of mitophagy/autophagy that in turn contributes to the removal of damaged mitochondria; this mechanism may be essential to overcome the effects of increased cellular levels of FA. Our work supports the existence of a pathway for targeted degradation of damaged mtDNA due to DSBs in a mitophagy/autophagy-dependent manner. Further studies are required to reveal factors involved in regulating this pathway.

## Supplementary information

Supplementary information

Supplementary information2

## Data Availability

All data generated or analyzed during the current studty are included.
