# Supplementary material for: Mitochondrial dysfunction and DNA damage accompany enhanced levels of formaldehyde in cultured primary human fibroblasts
Source: Sci Rep. 2020 Mar 27;10:5575. doi: 10.1038/s41598-020-61477-2 (PMC7101401; doi:10.1038/s41598-020-61477-2)

Mitochondrial dysfunction and DNA damage accompany enhanced levels of formaldehyde in cultured primary human fibroblasts

Cristina A. Nadalutti[1], Donna F. Stefanick[1], Ming-Lang Zhao[1], Julie K. Horton[1], Rajendra Prasad[1], Ashley M. Brooks[2], Jack D. Griffith[3] and Samuel H. Wilson[1*]

Supplemental Figure Legends

Supplemental Figure 1. Endogenous levels of FA in human primary fibroblasts. (a) Flow chart depicting strategy for measurement of FA levels in FSK cells after 24 h treatment. (b) Representative FA standard curve with known concentrations of FA between 0 μM and 40 μM. (c) FSK cells with (treated) and without (control) 250 μM FA after 24 h were collected, lysed and proteins were precipitated with TCA. Measurements of FA were conducted as described under Materials and Methods, and they are expressed as μM FA per milligram of extract protein. Experiments were performed in duplicate and repeated at least three times with different cell batches. In all panels, the bars represent the relative mean values and error bars S.E.M.

Supplemental Figure 2. OXPHOS genes regulated by enhanced intracellular FA in human primary fibroblasts. (a) Schematic overview of the electron respiratory chain complexes. (b) FA-regulated-OXPHOS-genes and relative fold change (FC). ND5 was the only mitochondrial-encoded-gene and COX8C with a very high FC, but with a p value that was not significant (*).

Supplemental Figure 3. Fusion and fission in mitochondrial human primary fibroblasts after 250 μM FA treatment for 24 h. (a-b) Flow cytometry quantification of fission (DRP1-616) and fusion (OPA-1) events in FSK cells with and without 250 μM FA for 24 h. The cells were collected and analysed by flow cytometry using antibodies specific for mitochondrial fission (DRP1-616-FITC) and fusion processes (OPA-1-FITC) conjugated with FITC. (b) Treatment with FA did not increase the proportion of fusion/fission events, when compared to the control groups (a). Experiments were performed in duplicate and repeated at least three times with different cell batches. Data were expressed as relative mean fluorescence intensity and in all panels, the bars represent the mean and error bars S.E.M.

Supplemenatal Figure 4. Accumulation of PINK1 in the mitochondria of human primary fibroblasts after mitochondrial depolarization. Representative confocal microscopy image of FSK cells following mitochondrial depolarization induced by CCCP at 1 μM final concentration. Mitochondria were visualized with TOMM20 (in red) and mitophagy (in green) with PINK-1. The nuclei were counterstained with DAPI (blue). Loss of $\Delta\Psi m$ induced accumulation of PINK-1 in the mitochondria. The colocalization of TOMM20 and PINK-1 (in yellow) is shown in the merged panel, as well as in the magnification yellow boxed areas on the right. Scale bar, 10 μm.

Supplemental Figure 5. FA-induced-DNA DSBs in the mitochondria of human primary fibroblasts. Representative confocal microscopy images of FSK cells following the indicated FA treatments, showing merged channels of DNA (red) and 53BP1 (green). The nuclei were counterstained with DAPI (blue). Scale bar, 10 μm. Magnifications of the yellow boxed areas in the right lower panels showing the colocalization of DNA and 53BP1 in yellow.

Supplemental Figure 6. Etoposide-induced-DNA DSBs in the mitochondria of human primary fibroblasts. (a-b) Representative confocal microscopy image of FSK cells following overnight incubation in the presence of 50 nM etoposide, showing DNA (in red) and γH2A.X (in green). The nuclei were counterstained with DAPI (blue). The colocalization of DNA and γ-H2A.X (in yellow) is shown in the merged panel and in the magnifications yellow box. Scale bar, 10 μm.

Supplemental Figure 7. Increased intracellular FA induced structural rearrangements in human primary fibroblasts. Replicate experiment showing representative EM micrographs of mitochondria from (a) untreated and (b) FSK treated cells with 250 μM FA for 24 h. Scale bar 1 μm.

Supplemental Figure 8. Increased intracellular FA induced selective accumulation of PINK1 in mitochondria of human primary fibroblasts. Figure 5a has been switched to a color-blind friendly palette showing merged channels of mitochondria visualized with TOMM20 (in magenta) and mitophagy (in light blue) with PINK-1. The nuclei were counterstained with DAPI (dark blue). Scale bar, 10 μm.

Supplemental Figure 9. Increased intracellular FA induced selective accumulation of PINK1 in mitochondria of human primary fibroblasts. (a) Replicate experiment showing representative confocal microscopy images of FSK cells following treatment with 250 μM FA for 24 h showing merged channels of mitochondria visualized with TOMM20 (in red) and mitophagy (in green) with PINK-1. The nuclei were counterstained with DAPI (blue). FA-induced accumulation of PINK-1 in mitochondria and the colocalization of TOMM20 and PINK-1 (in yellow) is shown in the merged panel as well as in the magnification yellow boxed aereas on the right. Scale bar, 10 μm. (b) Representative EM micrographs of autophagosomes from FSK cells (b) untreated and (c) treated with 250 μM FA for 24 h. Scale bar 2 μm.

Supplemental Figure 10. FA-induced-DNA-damage in human primary fibroblasts. Replicate experiment showing representative confocal microscopy images of FSK cells following the indicated FA treatments, displaying merged channels of TOMM20 (red) and γH2A.X (green). The nuclei were counterstained with DAPI (blue). Scale bar, 10 μm.

Supplemental Figure 11. FA-induced-DNA double strand breaks in the mitochondria of human primary fibroblasts. (a-b) Replicate experiment showing representative confocal microscopy images of FSK cells following the indicated FA treatments, presenting merged channels of DNA (in red) and γH2A.X (in green). The nuclei were counterstained with DAPI (blue). The colocalization of DNA and γ-H2A.X (in yellow) is shown in the merged panel and in the magnification yellow boxed areas with the green arrowheads indicating colocalization in the FA-treated group when compared to control FSK cells. Scale bar, 10 μm.

Supplemental Figure 1

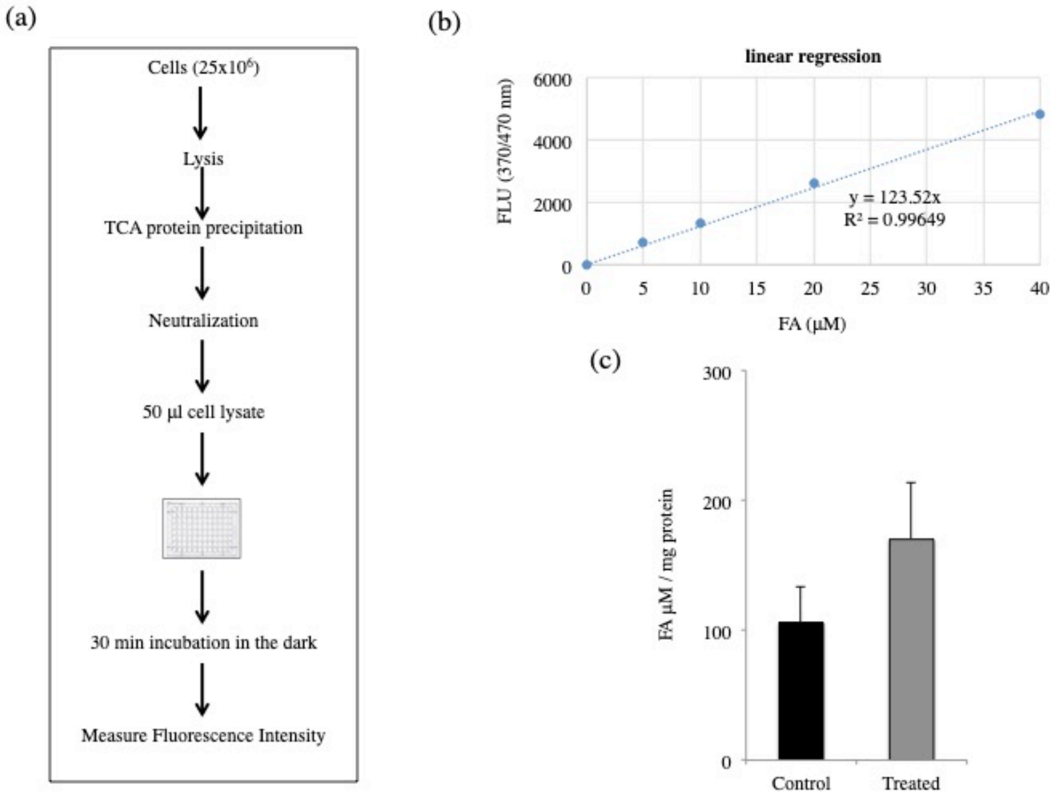

Supplemental Figure 2

(a)

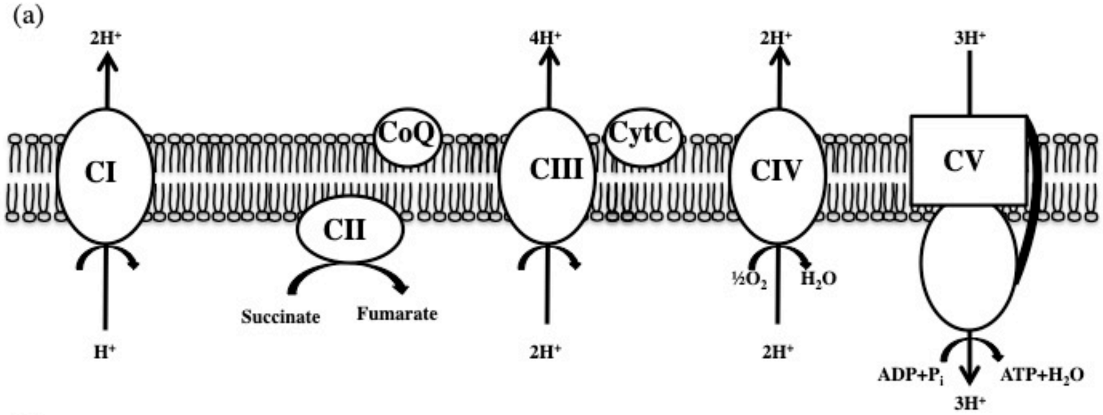

(b)

Complex I	FC
ND5	2.8137
NDUFV2	-1.9373
NDUFS1	-1.4542
NDUFA11	1.5005

Complex II	FC
SDHA	-2.4531

Complex III	FC
UQCRFS1	-1.6623

Complex IV	FC
COX8C	67.3934*
COX10	-2.4089

Complex V	FC
ATP5A1	-1.5551
ATP5D	1.7915

Supplemental Figure 3

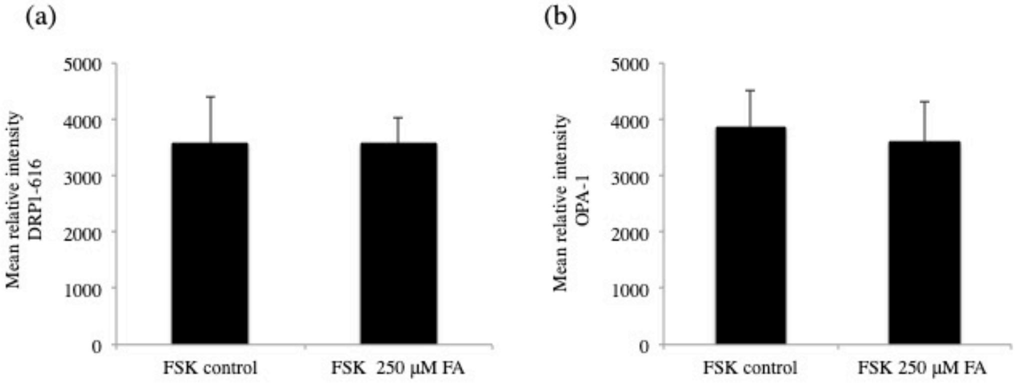

Supplemental Figure 4

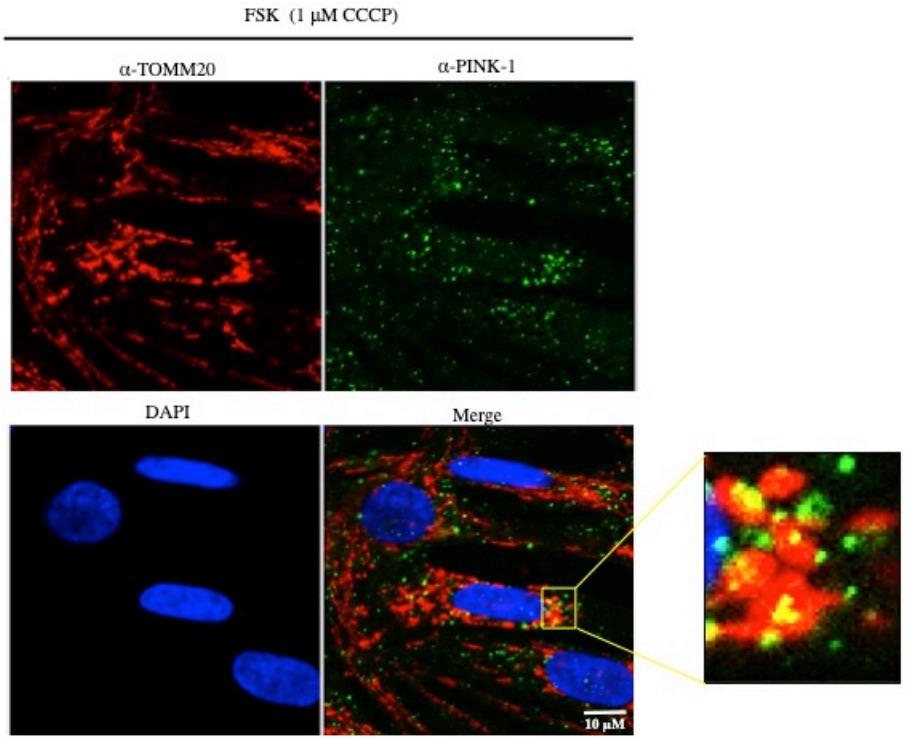

Supplemental Figure 5

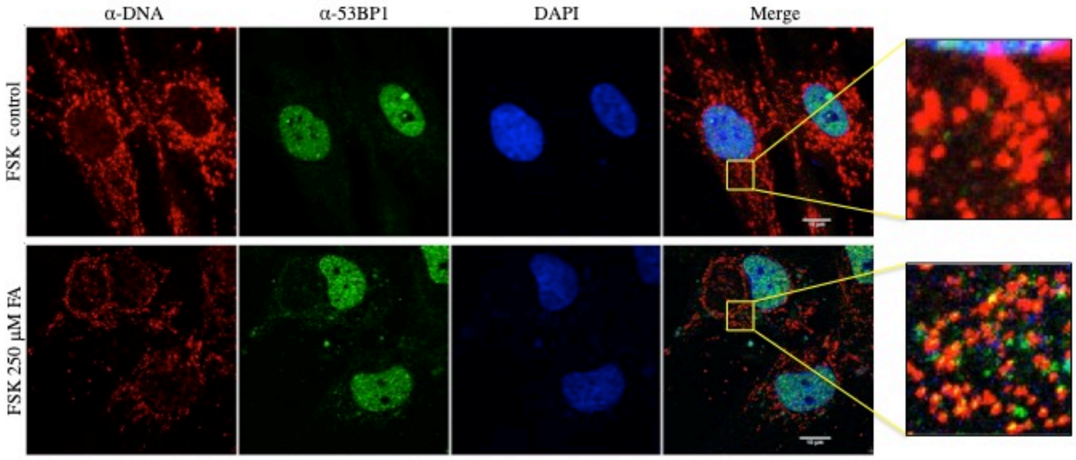

Supplemental Figure 6

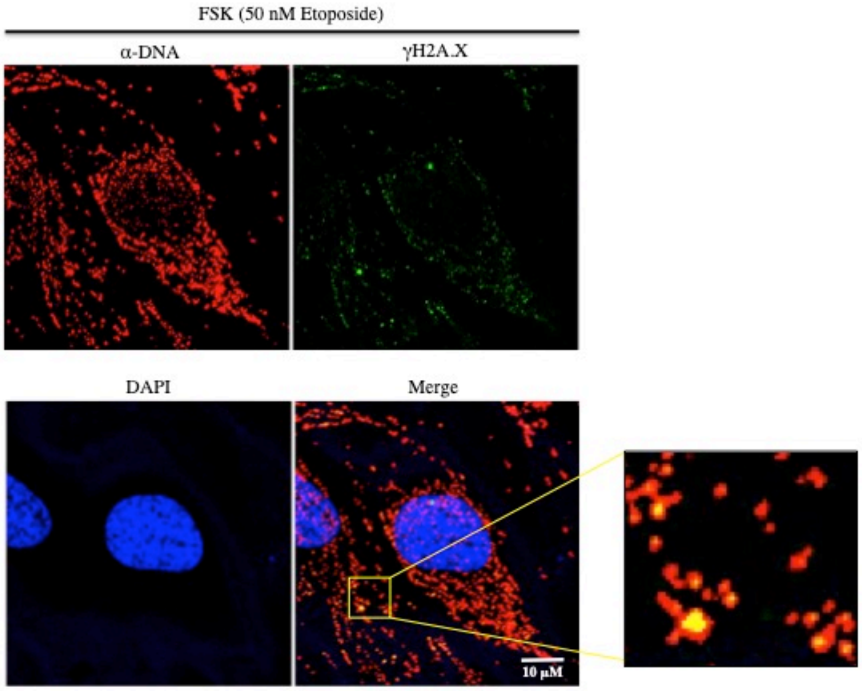

Supplemental Figure 7

Supplemental for Fig. 4: replicate

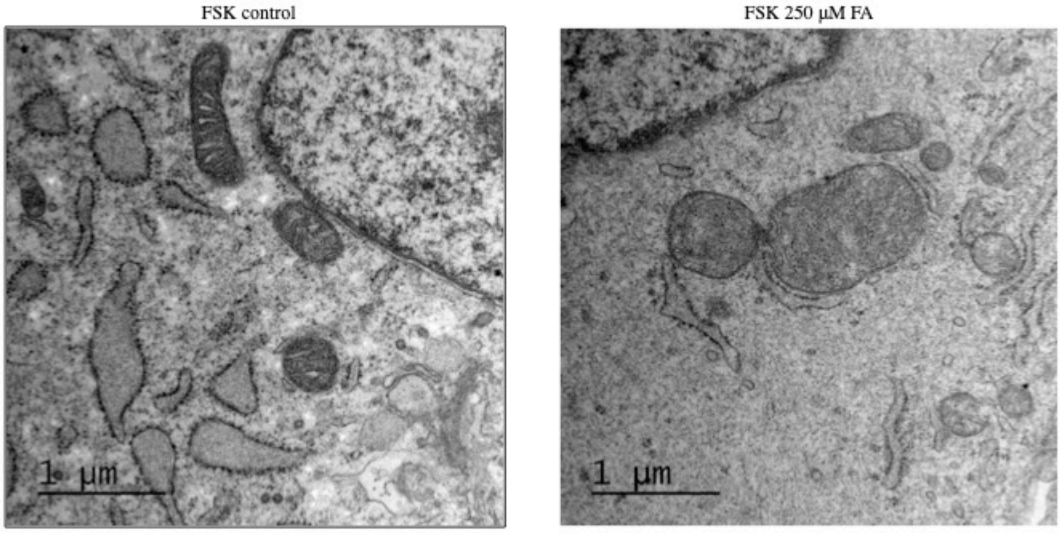

Supplemental Figure 8

Supplemental Fig. 5a: color blind friendly palette

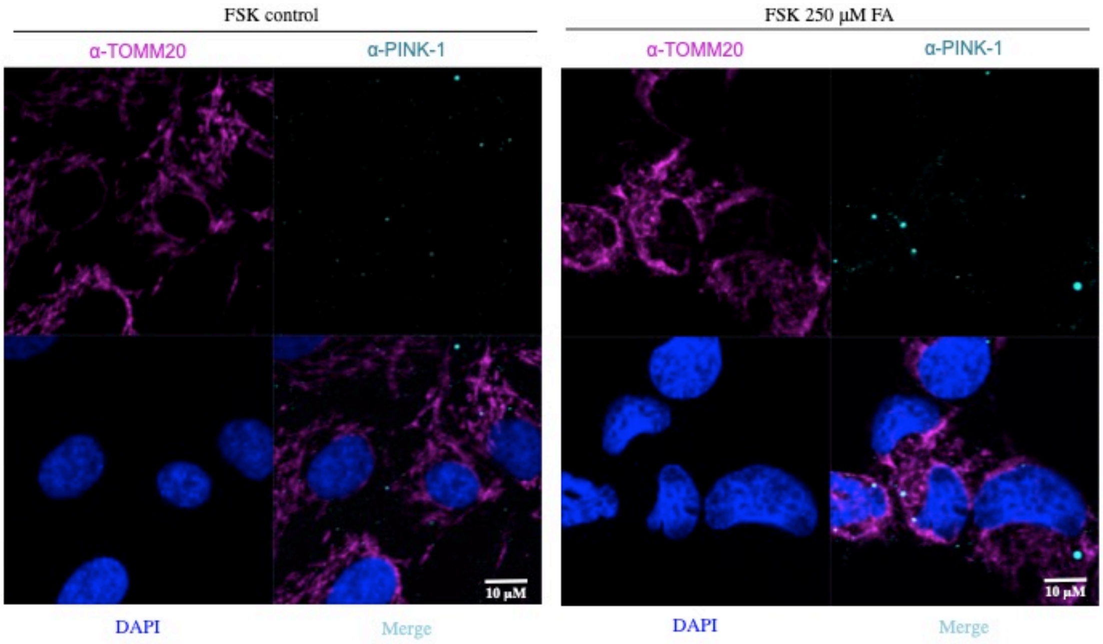

Supplemental Figure 9

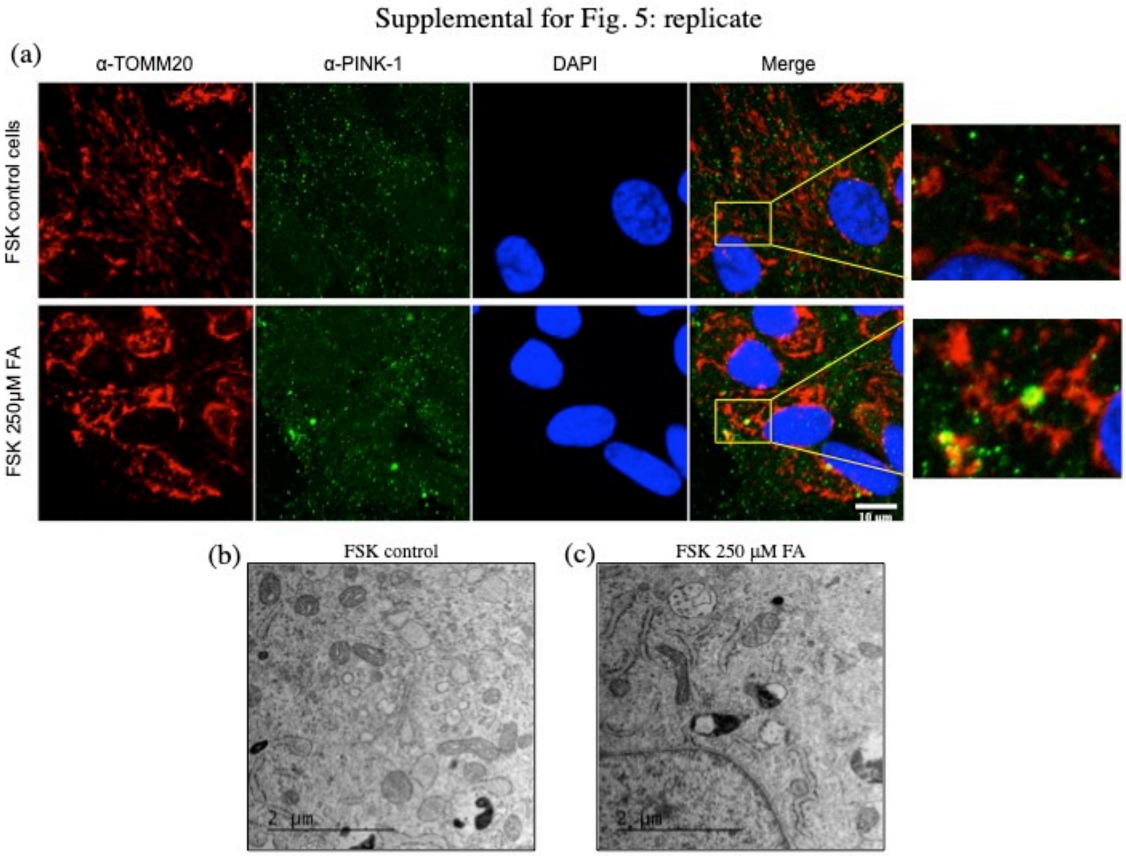

Supplemental Figure 10

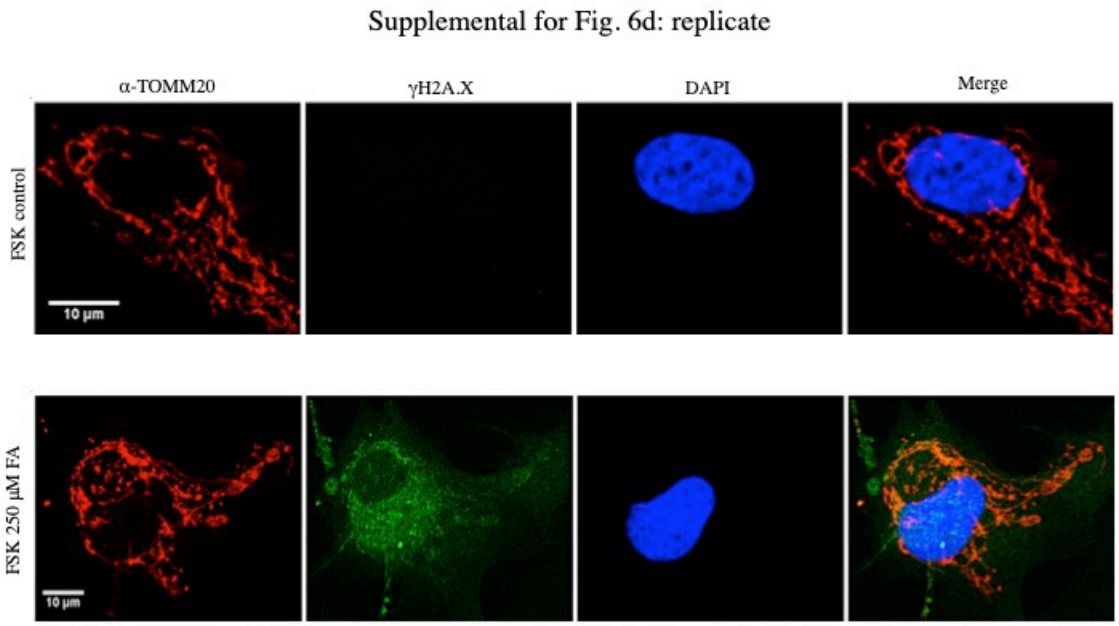

Supplemental Figure 11

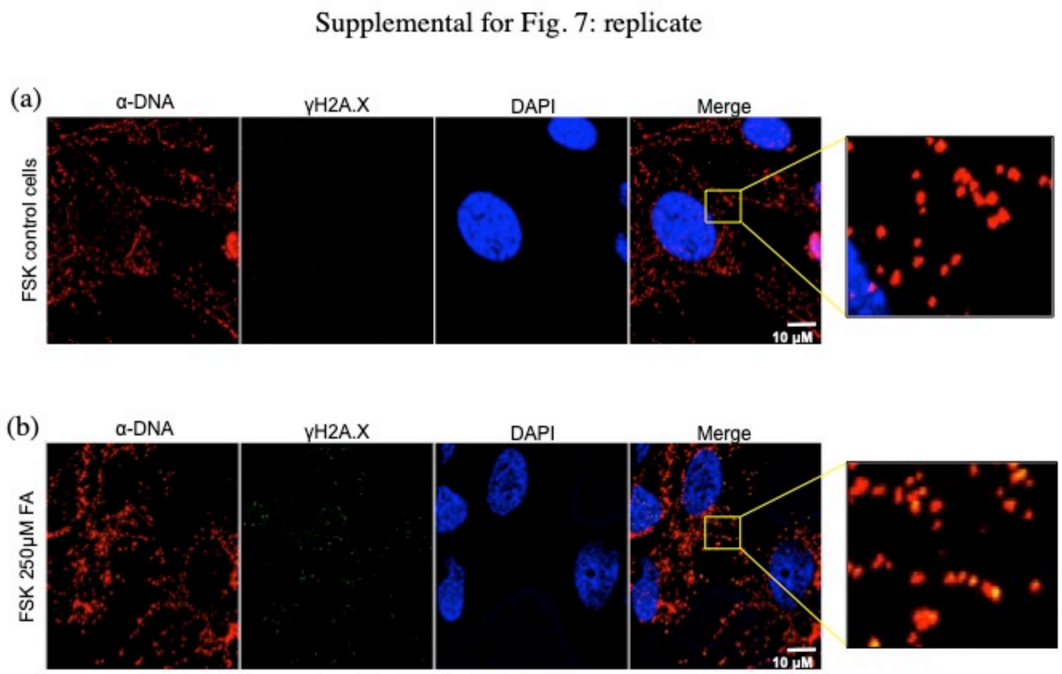

Supplement: Supplementary file 1 — Supplementary information [file 41598_2020_61477_MOESM1_ESM.pdf]
